# Cysteine-rich domain of scavenger receptor AI modulates the efficacy of surface targeting and mediates oligomeric Aβ internalization

**DOI:** 10.1186/1423-0127-20-54

**Published:** 2013-08-02

**Authors:** Fong-Lee Huang, Young-Ji Shiao, Sheue-Jane Hou, Cheng-Ning Yang, Yi-Jen Chen, Chao-Hsiung Lin, Feng-Shiun Shie, Huey-Jen Tsay

**Affiliations:** 1 Institute of Anatomy and Cell Biology, National Yang-Ming University, Taipei 11221, Taiwan; 2 National Research Institute of Chinese Medicines, Taipei 11221, Taiwan; 3 Department of Psychiatry, Cheng Hsin General Hospital, Taipei 112, Taiwan; 4 Institute of Neuroscience, Brain Research Center, School of Life Science, National Yang-Ming University, 155, Li-Nung Street, Section 2, Taipei 11221, Taiwan; 5 Department of Life Sciences, Institute of Genome Sciences National Yang-Ming University, Taipei 11221, Taiwan; 6 Division of Mental Health and Addiction Medicine, the Institute of Population Health Sciences, National Health Research Institutes, No. 35 Keyan Road, Zhunan Town, Miaoli County 350, Taiwan

**Keywords:** Scavenger receptor A, SRCR domain, Collagenous domain, Amyloid-β peptide, N- glycosylation

## Abstract

**Background:**

Insufficient clearance of soluble oligomeric amyloid-β peptide (oAβ) in the central nervous system leads to the synaptic and memory deficits in Alzheimer's disease (AD). Previously we have identified scavenger receptor class A (SR-A) of microglia mediates oligomeric amyloid-β peptide (oAβ) internalization by siRNA approach. SR-A is a member of cysteine-rich domain (SRCR) superfamily which contains proteins actively modulating the innate immunity and host defense, however the functions of the SRCR domain remain unclear. Whether the SRCR domain of SR-AI modulates the receptor surface targeting and ligand internalization was investigated by expressing truncated SR-A variants in COS-7 cells. Surface targeting of SR-A variants was examined by live immunostaining and surface biotinylation assays. Transfected COS-7 cells were incubated with fluorescent oAβ and acetylated LDL (AcLDL) to assess their ligand-internalization capabilities.

**Result:**

Genetic ablation of SR-A attenuated the internalization of oAβ and AcLDL by microglia. Half of oAβ-containing endocytic vesicles was SR-A positive in both microglia and macrophages. Clathrin and dynamin in SR-AI-mediated oAβ internalization were involved. The SRCR domain of SR-AI is encoded by exons 10 and 11. SR-A variants with truncated exon 11 were intracellularly retained, whereas SR-A variants with further truncations into exon 10 were surface-targeted. The fusion of exon 11 to the surface-targeted SR-A variant lacking the SRCR domain resulted in the intracellular retention and the co-immunoprecipitation of Bip chaperon of the endoplasmic reticulum. Surface-targeted variants were N-glycosylated, whereas intracellularly-retained variants retained in high-mannose states. In addition to the collagenous domain, the SRCR domain is a functional binding domain for oAβ and AcLDL. Our data suggest that inefficient folding of SR-AI variants with truncated SRCR domain was recognized by the endoplasmic reticulum associated degradation which leads to the immature N- glycosylation and intracellular retention.

**Conclusion:**

The novel functions of the SRCR domain on regulating the efficacy of receptor trafficking and ligand binding may lead to possible approaches on modulating the innate immunity in Alzheimer’s disease and atherosclerosis.

## Background

The accumulation of soluble oligomeric Amyloid-β peptide (oAβ) contributes to synaptic and memory deficits in Alzheimer’s disease (AD) [1]. The activation of microglia induced by oAβ is SR-A-dependent [2]. Previously, we identified SR-A as a prominent subtype of scavenger receptors mediating oAβ internalization in microglia by knockdown SR-A expression using siRNA [3]. In macrophages, SR-A mediates the internalization of low-density lipoprotein (LDL), leading to the formation of foam cells in atherosclerosis [4,5], and also mediates adhesion to the extracellular matrix [6]. Furthermore, SR-A regulates the induction of inflammatory cytokines in myocardial infarction and fungal infections [7]. In addition to its endocytotic activity, SR-A suppresses lipopolysaccharide-induced Toll-like receptor 4 signaling and nuclear factor-kappa B activation, thereby modulating the inflammatory response [8]. Knockout of SR-A reduces the lethality of septic shock and down-regulates TLR4 signaling in peritoneal macrophages [9]. Therefore, SR-A, a trimeric transmembrane glycoprotein, functions as a pattern recognition receptor and is actively involved in innate immunity and host defenses [10,11].

SR-A type I (SR-AI) contains six domains: a cytoplasmic domain, a transmembrane domain, a spacer region, an α-helical coiled-coil domain, a collagenous domain, and a C-terminal cysteine-rich (SRCR) domain encoded by exons 10 and 11. SR-AII and SR-AIII, alternative splicing isoforms of SR-AI, share all domains with SR-AI except for the SRCR domain [12]. SR-AII completely lacks the SRCR domain but binds the same ligands as SR-AI. However, SR-AIII, which has a truncated SRCR domain encoded by exon 11, is intracellularly retained. The cytoplasmic domain of SR-A is involved in cell adhesion and receptor internalization [13], with critical amino acids identified as being involved in SR-A surface targeting and interaction with signaling molecules [6,14]. Seven residues in the α-helical coiled-coil domain mediate the formation of the trimeric coiled-coil structure [15]. The collagenous domain mediates binding to the extracellular matrix [16], and point mutations in the positively charged lysine clusters in the SR-AII collagenous domain have been shown to decrease AcLDL binding activity [17].

Although more than 30 members of the SRCR superfamily have been identified, the function of the SRCR domain has remained unclear [18]. The expanding SRCR superfamily has been divided into two groups. Group A has an SRCR domain encoded by at least two exons with six cysteine residues, and group B has an SRCR domain encoded by a single exon with eight cysteine residues. Different members of the SRCR superfamily serve varying functions, including pathogen recognition and innate immune responses, and are associated with inflammation-related diseases, such as autoimmune diseases, atherosclerosis, and Alzheimer’s disease. SR-AI and MARCO (macrophage receptor with collagenous domain) are members of group A with highly conserved SRCR domains. Examination of the crystal structure of the mouse MARCO SRCR domain revealed that the monomeric recombinant SRCR domain is a compact, globular domain [19]. The SRCR domain of MARCO was identified as the binding domain for bacteria, acetylated-LDL (AcLDL), and the extracellular matrix [19,20]. The function of the SRCR domain of SR-AI, however, remains unclear. The SRCR domain of several group B members, including CD163, Spα, and S5DSRCRB, functions as the binding domain for haptoglobin-hemoglobin complexes, lipopolysaccharide, and bacteria and modulates innate immunity in macrophages [21-23].

In the present study, we identified critical roles of the SRCR domain play in SR-AI surface trafficking and internalization of oAβ and AcLDL. Our results provide insight into the critical role of the SRCR domain in N-glycosylation and receptor surface targeting of SR-AI, which is a prerequisite for the uptake of oAβ and AcLDL by microglia and macrophages in the initiation stage of AD and atherosclerosis.

## Methods

### Materials

Aβ1-42 and fluorescein amidite (FAM)-labeled Aβ1-42 were purchased from American Peptide (Sunnyvale, CA) and Biopeptide (San Diego, CA). Antibodies against Aβ were purchased from Signet (Dedham, MA). Anti-BiP antibody, Alexa-labeled AcLDL, and Lipofectamine 2000 were purchased from Invitrogen (Carlsbad, CA). Rat anti-mouse SR-A and rabbit anti-human SR-A were purchased from AbD Serotec (Oxford, United Kingdom) and Santa Cruz (California, USA). Alexa Fluor488-conjugated secondary antibody was purchased from Molecular Probe (Oregan, USA). Sulfo-NHS-SS-biotin and NeutrAvidin were purchased from Pierce (Rockford, IL). PNGase F and Endo H were purchased from New England BioLabs (Ipswich, MA).

### Cell cultures and transfection

Human macrophages were prepared as previously described [24]. Briefly, whole blood from healthy donors was fractionated through a Histopaque-1077 density gradient (Sigma-Aldrich, St. Louis, MO). The mononuclear cells were re-suspended in serum-free RPMI 1640 medium and differentiated into macrophages by incubation with 10 ng/mL human macrophage colony stimulating factor (R&D, Minneapolis, MN) for 7 days. Primary mouse microglia were prepared as previously described [3]. Briefly, cortices from newborn wild-type and SR-A homozygous knockout mouse pups were dissociated and grown in DMEM with 10% low-endotoxin FBS. Microglia were isolated from the mixed glia after 14 days. COS-7 cells, fibroblast-like cells derived from monkey kidney tissue and J774 cells, macrophage cells were maintained in Dulbecco’s Modified Eagle Medium (DMEM) containing 10% heat-inactivated fetal bovine serum at 37°C in a 5% CO_2_ humidified atmosphere. THP-1 cell is a human monocyte cell line, which were differentiated into macrophages by phorbol 12-myristate 13-acetate (PMA). Human SR-AI cDNA was provided by Dr. Qi Chen (Nanjing Medical University, Nanjing, China). The sequences of primers and ligation sites used to construct SR-AI variants are shown in Additional file 1: Table S1. Site-directed mutagenesis was performed using the QuikChange site-directed mutagenesis kit (Stratagene, La Jolla, CA). COS-7 cells (3 × 10^5^) were transfected with 2 μg SR-AI or variants per well in 6-well plates using Lipofectamine 2000 according to manufacturer instructions. After 24 h, cells were subjected to ligand binding, surface protein biotinylation, and immunocytochemical analyses. The involvement of clatherin in the internalization of oAβ was assessed by cotransfecting with SR-A clatherin shRNA for 48 h. The internalization of oAβ was performed after cotransfecting HA-tag dynamin 2 dominant negative (K44A-dyn) with SR-A for 24 h.

### Live immunostaining and immunocytochemistry

To detect surface-targeted SR-A, live transfected COS-7 cells were incubated with rabbit anti-human SR-A antibody at 1:500 dilution, followed by incubation with secondary antibody conjugated to Alexa Fluor 488. To detect cytosolic SR-A, permeabilized mouse microglia were incubated with rat anti-mouse SR-A antibody. Permeabilized human macrophages and transfected COS-7 cells were incubated with rabbit anti-human SR-A antibody, followed by incubation with secondary antibody conjugated to Alexa Fluor 594. Coverslips were mounted with Vitashield (Vector Laboratories, Peterborough, Cambridgeshire, UK) and images were taken using a confocal microscope (Olympus, FV-1000 and FV-10i). The experiments were repeated at least three times.

### Ligand binding and internalization

FAM-labeled oAβ was prepared and biochemically characterized as described [3,25]. Prior to each usage, a liquate of oAβ was centrifuged at 14,000 g at 4°C for 10 min to remove fibrillar and aggregated Aβ. Cells were incubated with 2 μM FAM-oAβ on ice for 30 min and live immunostained with anti-SR-A antibody for another 30 min. Then, cells were fixed with 4% paraformaldehyde to assess the oAβ binding ability at the plasma membrane. To assess the levels of internalized oAβ and AcLDL, transfected cells were incubated with 1 μM FAM-oAβ for 1 h or 5 μg/mL Alexa 594-labeled AcLDL for 1.5 h in serum-free DMEM at 37°C. Then, cells were fixed with 4% paraformaldehyde and immunostained with anti-SR-A antibody. Images were taken using a confocal microscope. The fluorescence intensities of more than 100 SR-A-positive cells in five random fields were analyzed using MetaMorph software (ver. 7.1; Molecular Devices).

### Surface biotinylation

Surface proteins were labeled with Sulfo-NHS-SS-biotin following manufacturer instructions (Pierce, Rockford, IL). Briefly, cells were incubated with membrane-impermeable sulfo-NHS-SS-biotin on ice for 30 min. Unbound biotin was quenched with Tris buffer on ice for 10 min. Cells were lysed with NP40-containing lysis buffer (Invitrogen, Life Technologies AS, Norway) and incubated with NeutrAvidin beads overnight at 4°C. Bound proteins were eluted from the NeutrAvidin beads by boiling. After centrifugation, the supernatants were used in subsequent analyses.

### Peptide N-glycosidase (PNGase F) and endoglycosidase (Endo H) cleavage

The N-glycosylation status of SR-AI and variants was determined by incubating with PNGase F or Endo H following manufacturer instructions. Briefly, glycoprotein denaturing buffer was added to the total cell lysates and surface biotinylated lysates. After boiling for 10 min, the mixtures were incubated with PNGase F (500 U) or Endo H (1000 U) for 18 h at 37°C. The protein was boiled for 10 min and subjected to SDS gel electrophoresis.

### Western blot analysis and immunoprecipitation

After electrophoresis, proteins were transferred onto PVDF membranes. After blocking, the membranes were incubated with anti-SR-A antibody at 1:1,000 dilution, transferrin receptor antibody at 1:500 dilution, and β-actin antibody at 1:5,000 dilution. After incubation with the secondary antibody, immune complexes were detected using an enhanced chemiluminescence kit (GE Healthcare, Burlingame, CA). The luminescence intensity was quantified using densitometry. The experiments were repeated at least three times. For immunoprecipitation, cells were lysed in lysis buffer containing protease inhibitor cocktail (Sigma-Aldrich, St. Louis, MO). Rabbit anti-human SR-A antibody were coupled to paramagnetic Dynabead protein G (Invitrogen, Life Technologies AS, Norway). Lysates were incubated with the antibody-Dynabead complex overnight at 4°C. The immune complex was subjected to Western blot analysis using anti-BiP antibody at 1:1,000 dilution. The Western blot was incubated with anti-SR-A antibody served as a loading control (data not shown). The experiments were repeated at least three times.

### Statistical analysis

**Figure 1:**
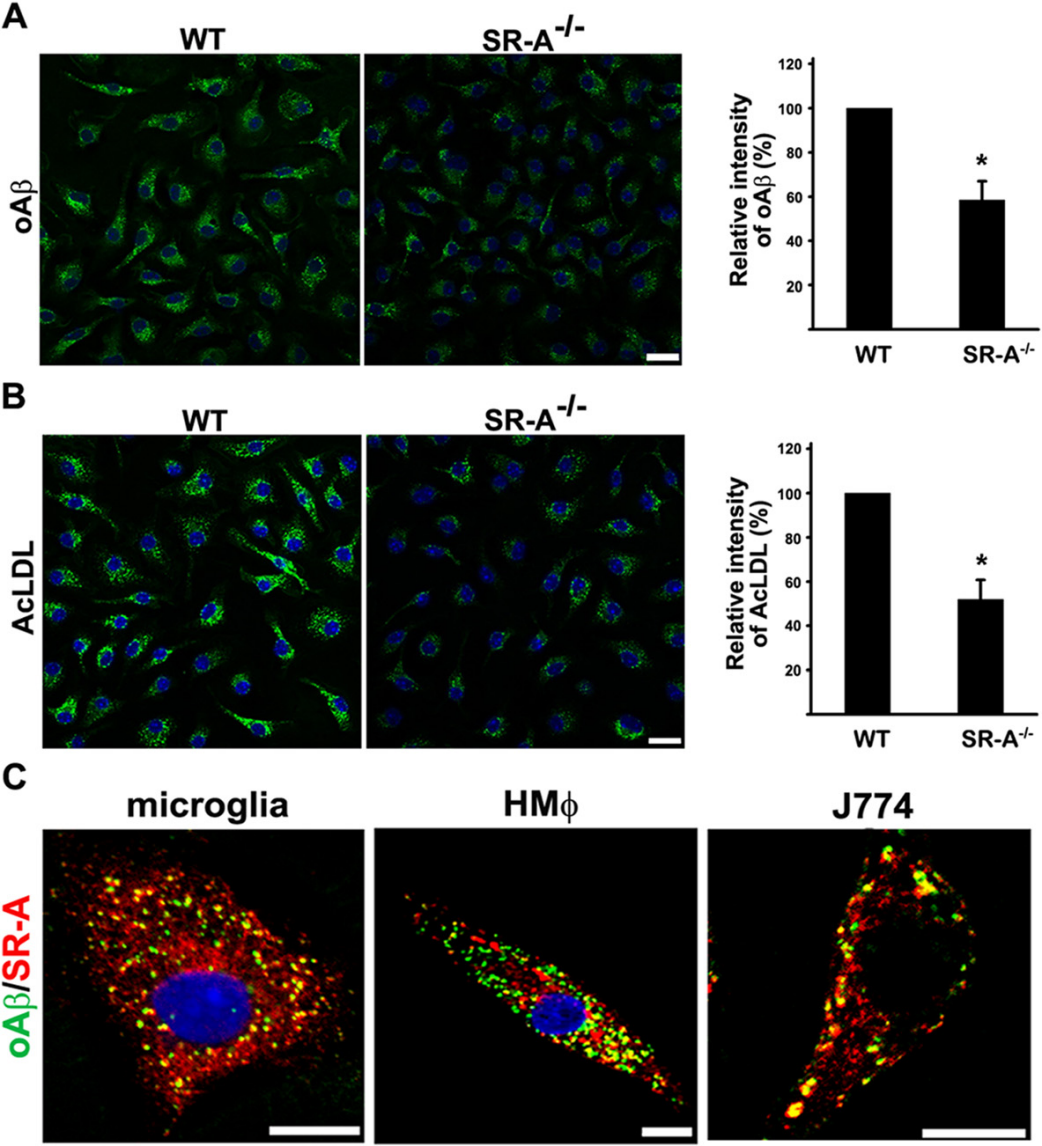
**Microglia of SR-A knockout mice internalizes less oAβ and AcLDL. A** and **B**, Primary microglia isolated from wild-type (WT) and SR-A knockout mice were incubated with fluorescent oAβ and AcLDL. Representative confocal images and quantification of relative fluorescence intensities of internalized oAβ and AcLDL showed that less internalization of oAβ and AcLDL by microglia of SR-A knockout mice. Scale bar, 20 μm. More than 60 cells were analyzed. Bars indicate mean ± SEM of three independent experiments (*p < 0.05). **C**, Primary microglia, human blood-derived macrophages (HMϕ), and J774 cells were incubated with FAM-oAβ and immunostained with anti-SR-A antibody. Representative confocal images showed that oAβ was highly co-localized with SR-A in endocytic vesicles. The experiments were repeated at least three times. Scale bar, 10 μm.

**Figure 2:**
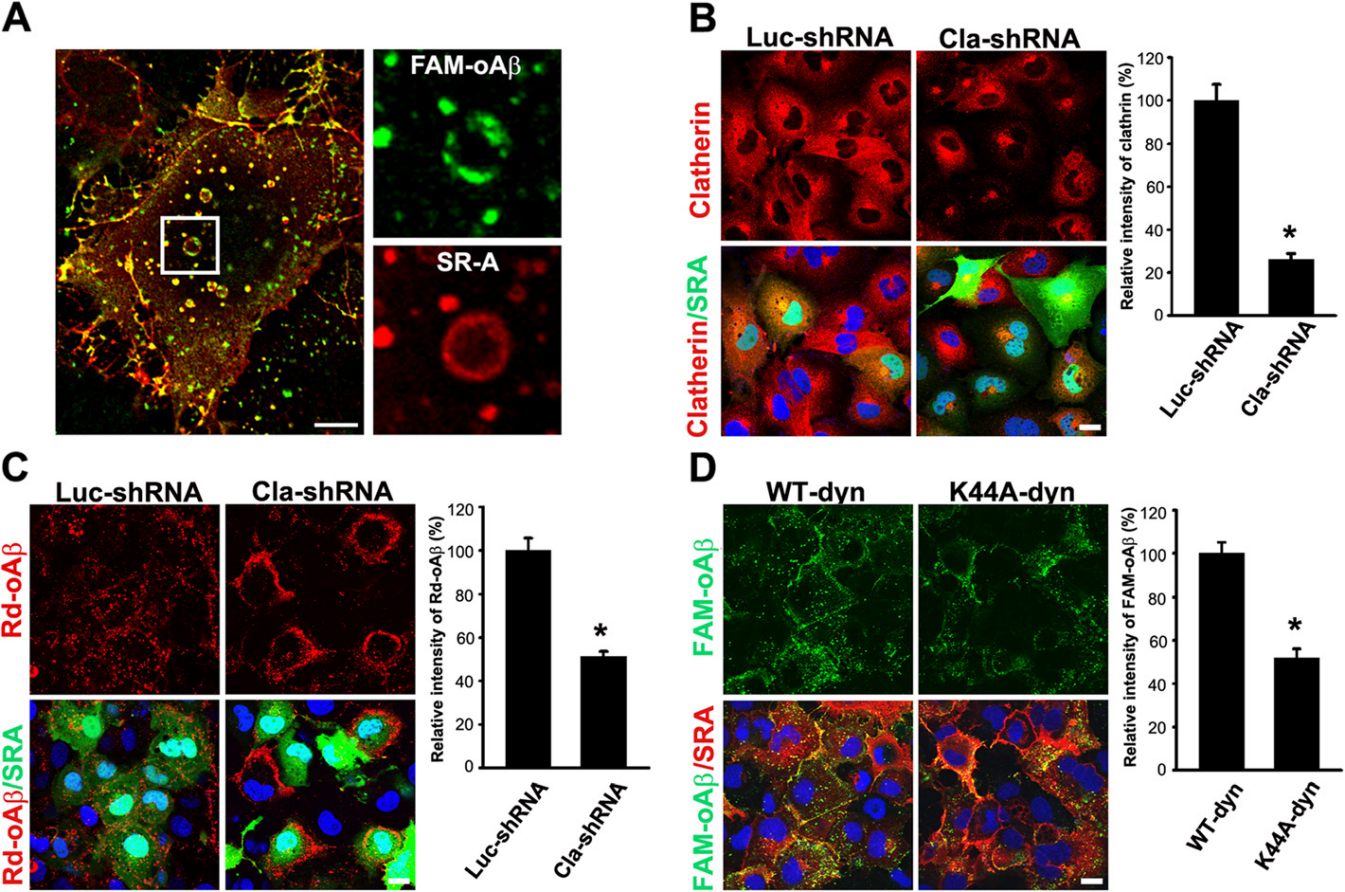
**Clathrin and dynamin-2 mediate SR-AI-dependent oAβ internalization. A**, SR-AI-transfected COS-7 cells were incubated with FAM-oAβ (green) and immunostained with anti-SR-A antibody (red). Representative confocal images showed the co-localization of FAM-oAβ and SR-AI in endocytotic vesicles (left panel). **A** magnified image of the boxed region was shown in the right panels. Scale bar, 10 μm. **B**, COS-7 cells were co-transfected with SR-AI and clathrin shRNA or luciferase shRNA (negative control). The confocal images and the quantification of clathrin immunoreactivity showed clathrin shRNA knockdown the expression of clatherin in SR-AI-positive cells. The experiments were repeated at least three times. **C**. The relative level of internalized oAβ was significantly reduced by clathrin shRNA compared with luciferase shRNA in SR-AI-positive cells. **D**, COS-7 cells were co-transfected with SR-AI and HA-tagged wild type dynamin-2 or dominant-negative dynamin-2 (K44A). The relative intensity of internalized oAβ was significantly reduced by dynamin-2 (K44A) in SR-AI-positive cells. More than 100 SR-AI-positive cells were analyzed. Bars indicate mean ± SEM of three independent experiments (*p < 0.05).

All data were expressed as mean ± standard error of the mean (SEM) and analyzed by one-way analysis of variance followed by Tukey’s HSD *post hoc* tests using SPSS 11.5 software (SPSS Inc., Somers, NY). Values of *p* < 0.05 were considered statistically significant. Experimental groups labeled with different letters were significantly different from each other. Experimental groups labeled with identical letters were not significantly different from each other. In Figures 1 and 2, asterisks represent statistically significant differences.

## Results

### Genetic ablation of SR-A attenuated the internalization of oAβ and AcLDL by primary microglia

The role of SR-A in oAβ internalization was examined using microglia isolated from SR-A knockout mice. The level of internalized oAβ and AcLDL by microglia isolated from SR-A knockout mice was significantly reduced compared with that of microglia isolated from wild-type mice (Figure 1A,B). The percentage of oAβ and SR-A-positive endocytic vesicles in primary mouse microglia, human monocyte-derived macrophages, and macrophage cells J774 were 49.1 ± 3.1, 46.21 ± 9.2 , and 56.5 ± 6 (Figure 1C). In addition to SR-A, our data also suggested that there are the other receptors mediating oAβ internalization in microglia and macrophage [26-28].

### Clathrin and dynamin 2 are involved in SR-AI-mediated oAβ internalization

COS-7cells are commonly used for the functional study of SR-A [29,30]. The N-glycosylation status of transfected human SR-AI in COS-7 cells mimics endogenous human SRA of human blood-derived macrophage and PMA-differentiated THP1 cells (Additional file 2: Figure S1A). COS-7 cells cannot internalize Aβ and AcLDL, were used to characterize the functions of individual domain of human SR-AI (Additional file 2: Figure S1B and C). The internalized Aβ was colocalized with SR-AI in endocytotic vesicles in SR-AI-transfected COS-7 cells (Figure 2A). The involvement of clathrin and dynamin 2 in SR-AI-mediated oAβ internalization was examined by cotransfecting SR-AI with clathrin shRNA or a dominant-negative mutant of dynamin 2 (k44A-dyn). The expression of clathrin was effectively knockdown by clathrin shRNA (Figure 2B). The level of internalized oAβ was significantly reduced by clathrin shRNA (Figure 2C). OAβ was retained at the plasma membrane of clathrin shRNA and SR-AI cotransfected cells. It has been shown that receptor-mediated endocytosis is dependent on dynamin [31]. The overexpression of wild-type dynamin-2 did not affect oAβ internalization (Figure 2D). However, the overexpression of k44A-dyn in SR-A in COS-7 cells, inhibited oAβ internalization. The level of internalized oAβ in SR-AI-positive COS-7 cells was significantly reduced by k44A-dyn. Thus, our data suggested that clathrin and dynamin 2 were involved in SR-AI-mediated oAβ endocytosis.

### The SRCR domain of SR-AI is critical for receptor surface targeting

**Figure 3:**
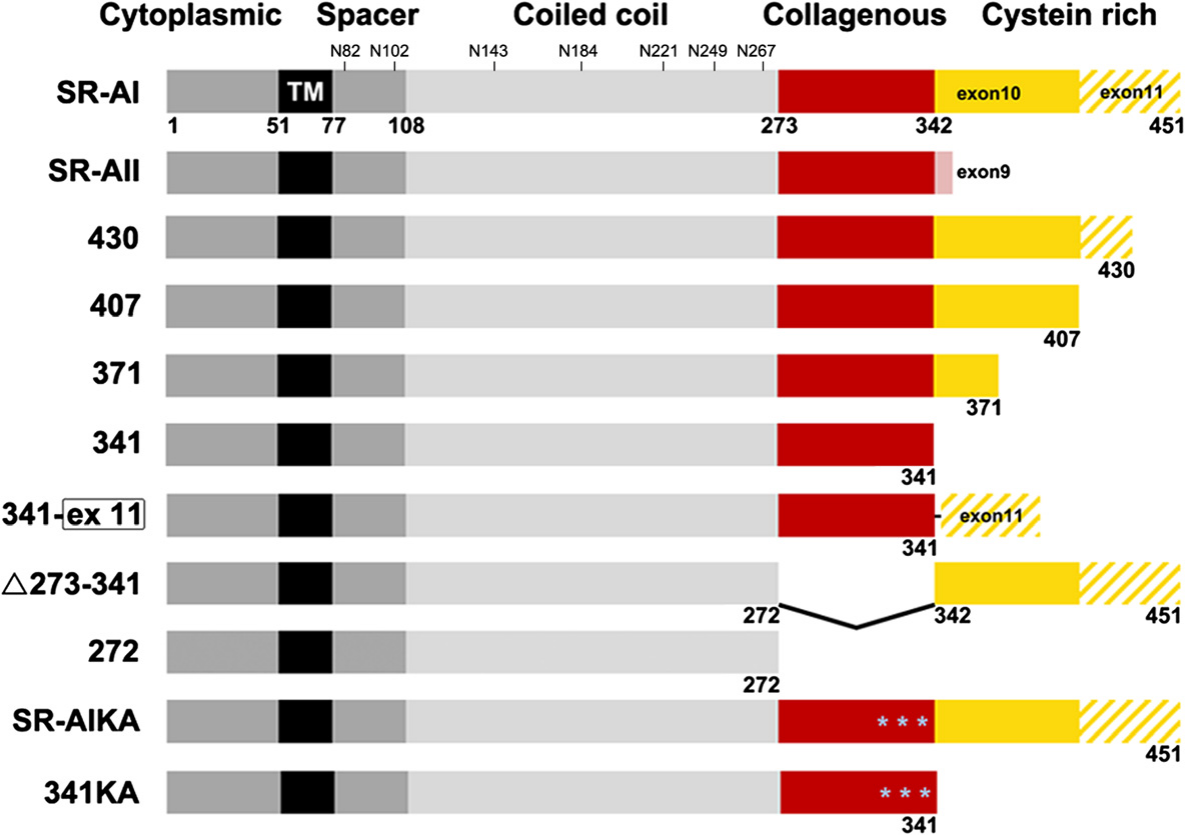
**Schematic representation of human SR-AI, SR-AII, and SR-AI variants.** Constructs 430, 407, 371, and 341 were SR-AI variants with truncated SRCR domains. Construct 341wirh exon 11 fused to the C-terminus of the collagenous domain mimicking SR-AIII is designated as 341-ex 11. * indicates three lysine residues in the collagenous domain that were mutated to alanine at 332, 335, 338 in 341KA and SR-AIKA. Seven putative N-glycosylation sites in the spacer and coiled coil domains of SR-AI were labeled.

**Figure 4:**
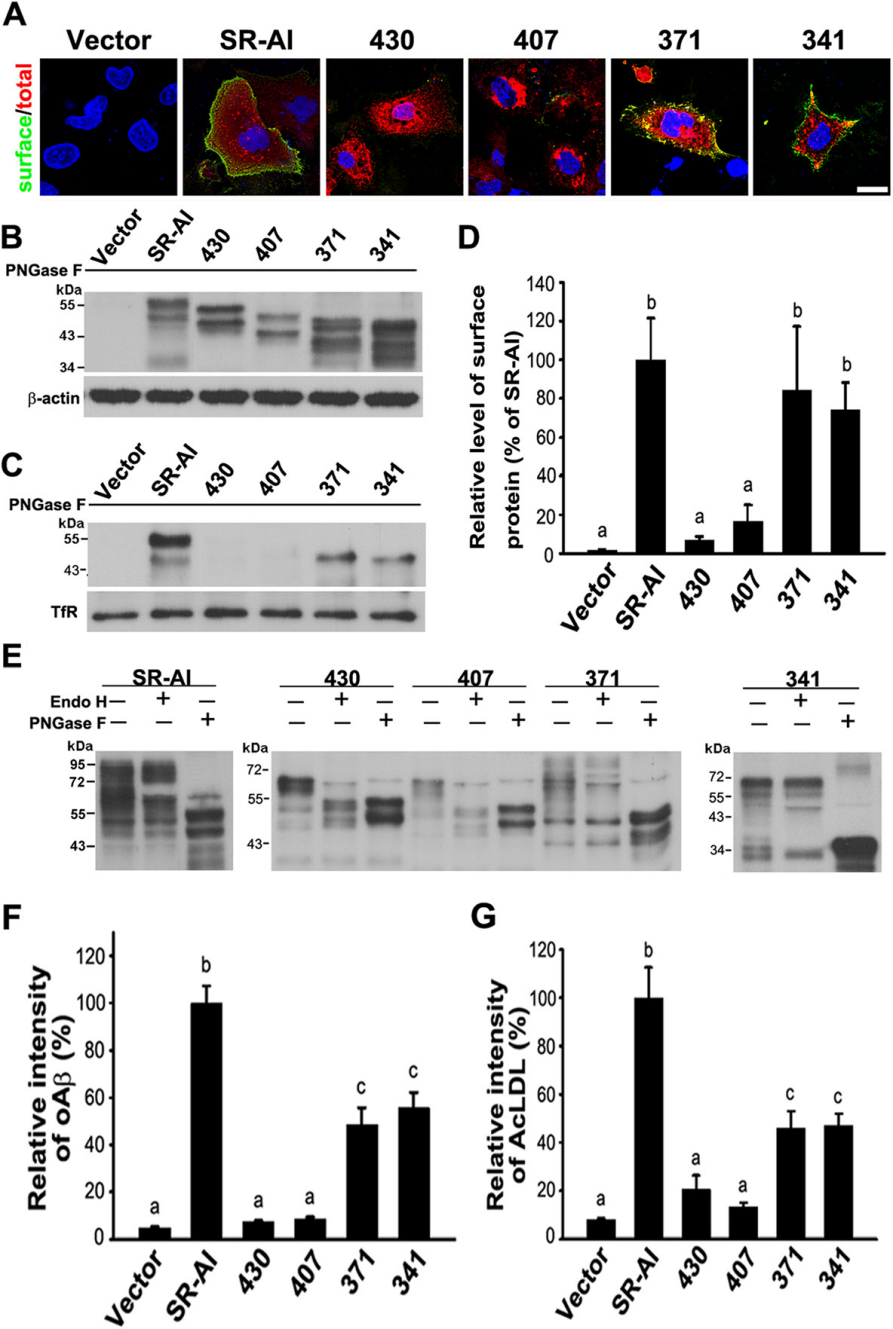
**The SRCR domain is critical for the surface targeting and N-glycosylation of SR-AI.** COS-7 cells were transfected with SR-AI and variants with truncated SRCR domains. **A**, Surface-targeted SR-AI variants were detected by live immunostaining (green). Cytosolic SR-AI variants were detected by immunocytochemistry (red). The yellow signal in the merged confocal images indicated that SR-AI, 371, and 341 were surface-targeted. Nuclei were counterstained with Hoechst 33258 (blue). Scale bar, 20 μm. **B** and **C**, Western blot analysis of total cell lysates and avidin pull-down of biotinylated lysates after PNGase F cleavage. β-actin and transferrin receptor (TfR) served as loading controls. **D**, Relative levels of surface-targeted SR-AI variants were quantified by densitometry. **E**, Western blot analysis of total cell lysates after PNGase F or Endo H cleavage. **F** and **G**, Transfected cells were incubated with fluorescent oAβ and AcLDL followed by immunostaining using anti-SR-A antibody. The experiments were repeated at least three times. Relative fluorescence intensities of internalized oAβ and AcLDL for more than 100 SR-AI-positive cells were quantified using MetaMorph software. Bars indicate mean ± SEM of three independent experiments. Experimental groups labeled with different letters (a, b, c) were significantly different from each other (*p* < 0.05).

Next, we assessed the role of the SRCR domain in the protein trafficking of SR-AI by expressing mutated variants with serial truncations of the SRCR domain in COS-7 cells (Figure 3). The comparable enzymatic activities of co-transfected β-galactosidase across variants suggest that their transfection efficiencies were similar (data not shown). Merged confocal images of live immunostaining and immunocytochemistry showed that full-length SR-AI and deletion variants 371 and 341 were surface-targeted, whereas deletion variants 430 and 407 were retained intracellularly (Figure 4A). The molecular weight of nascent SR-AI is approximately 50 kDa. In the total cell lysates while SR-AI was in the process of protein modifications, a diffuse block was detected by Western bolt analysis. To quantify the expression level of SR-A variants, cell lysates were incubated with PNGase F, which cleaves N-acetylglucosamine from asparagine at N-glycosylation sites. In SR-AI-transfected cell lysate, we detected one major band at 55 kDa and a second band close to 50 kDa (Figure 4B). To investigate the identity of these bands, we performed tandem mass spectrometry analyses after enriching the proteins by immunoprecipitation (data not shown). Although we found that these two bands exhibited partial SR-A sequences, our data was not sufficient to determine the cause of the two bands detected in the cell lysates after PNGase F cleavage. The expression levels of SR-AI variants in the total cell lysates were comparable.

To quantify the level of surface-targeted SR-AI and variants, the surface protein biotinylation assay was performed. Avidin pull-down of biotinylated lysates was subjected to PNGase F cleavage and Western blot analysis. One major band at 55 kDa and one minor band close to 50 kDa were only detected in SR-AI-, 371-, and 341-transfected cell lysates, suggesting that SR-AI, 371, and 341 were surface-targeted and that 430 and 407 were intracellularly retained (Figure 4C,D).

Endo H cleaves N-glycans in a high-mannose state and but not complex N-glycans. It has been shown that the complex N-glycan of SR-AI is Endo H-resistant, whereas the N-glycan of SR-AIII, which is in a high-mannose state, is Endo H-sensitive [12]. In total cell lysates, SR-AI and variants were undergoing N-glycosylation at different states, therefore a diffused block of signal was detected (Figure 4E). The molecular weight shift by PNGase F cleavage of SR-AI variants suggests that SR-A variants were N-glycosylated. SR-AI, 371, and 341 were predominantly Endo H-resistant, but 430 and 407 were Endo H-sensitive, suggesting that the deletion of exon 11-encoded portion of the SRCR domain alters their N-glycosylation status. The amount of oAβ and AcLDL internalized by 371- and 341-positive cells was significantly lower than that internalized by full-length SR-AI. However, the levels of internalized oAβ and AcLDL in variants 430 and 407 were not different from that of the vector-only. These results suggest that the SRCR domain plays critical roles in the protein trafficking and ligand internalization (Additional file 3: Figure S2A,B and Figure 4F,G).

### The fusion of exon 11 of SRCR domain with the surface-targeted SR-A variant mimics the intracellular retention of SR-AIII

**Figure 5:**
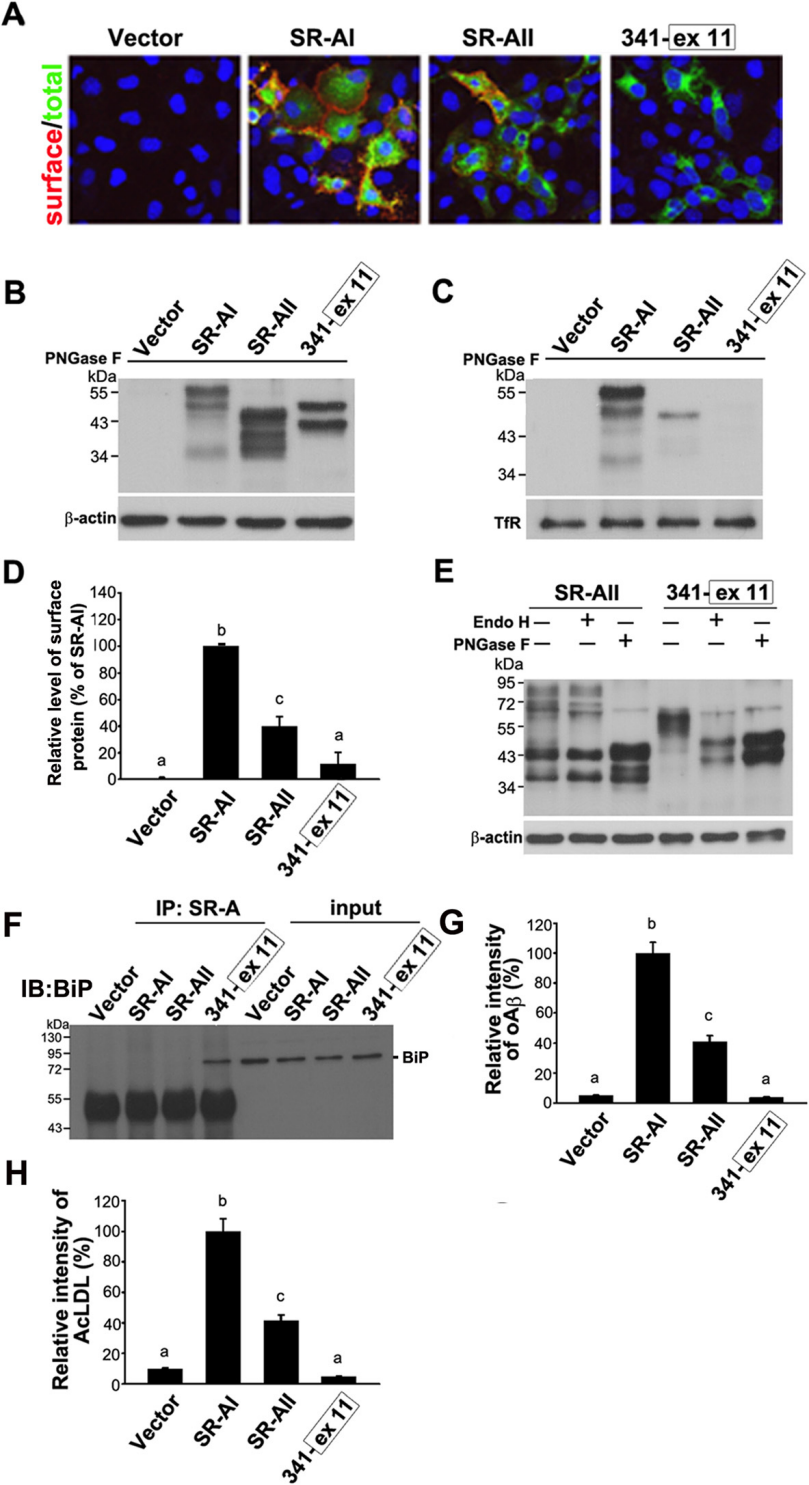
**The surface targeting and ligand internalization are abolished by fusing of exon 11 with SR-AI variant 341. A**, Surface-targeted SR-A variants were detected by live immunostaining (red). Cytosolic SR-A variants were detected by immunocytochemistry (green). The yellow signal in the merged confocal images indicated that SR-AI and SR-AII were surface-targeted. Nuclei were counterstained with Hoechst 33258 (blue). **B** and **C**, Western blot analysis of total cell lysates and avidin pull-down of biotinylated lysates after PNGase F cleavage. **D**, Relative levels of surface-targeted SR-AI variants were quantified by densitometry. **E**, Western blot analysis of total cell lysates after PNGase F or Endo H cleavage. **F**, Lysates of COS-7 cells were immunoprecipitated with anti-SR-A antibody and subjected to Western blot analysis using anti-BiP. The experiments were repeated at least three times. **G** and **H**, Transfected cells were incubated with fluorescent oAβ and AcLDL followed by immunostaining using anti-SR-A antibody. Relative fluorescence intensities of internalized oAβ and AcLDL for more than 100 SR-A-positive cells were quantified using MetaMorph software. Bars indicate mean ± SEM of three independent experiments. Experimental groups labeled with different letters were significantly different from each other (*p* < 0.05).

SR-AIII, the splicing isoform of SR-AI with a truncated SRCR domain encoded by exon 11 is intracellularly retained. We have shown that 341 with the collagenous domain only was surface-targeted. The effect of fusing exon 11 with 341 mimicking SR-AIII was examined next. The confocal images of immunostaining confirmed that 341-exon11 was intracellularly retained (Figure 5A). The expression levels of SR-AI, SR-AII, and 341-exon11 in the total cell lysates were comparable (Figure 5B). The surface protein biotinylation assay showed that 341-exon11 was not targeted to the plasma membrane (Figure 5C,D). The surface level of SR-AII was significantly lower than that of SR-AI. Surface-targeted SR-AI and SR-AII were predominantly Endo H-resistant, whereas 341-exon11 was Endo H-sensitive (Figure 5E). It indicated that the fusing of exon 11 with 341 attenuated its N-glycosylation and surface targeting.

BiP is an important protein chaperone for protein quality control in the endoplasmic reticulum (ER). Prolonged binding of BiP can trigger the dislocation of misfolded proteins from the ER into the cytoplasm for degradation [32]. An immunoprecipitation assay was performed by incubating total lysates of SR-AI-, SR-AII-, 341-exon11-, and vector-transfected cells with anti-SR-A antibody. After eluting from anti-SR-A antibody-conjugated beads, protein was subjected to Western blot analysis using anti-BiP antibody (Figure 5F). BiP was detected in all of input lysates, however, BiP was only co-immunoprecipitated with 341-exon11. This suggested that the fusion of exon 11 to 341 resulted in the prolonged binding of BiP. Consistently, SR-AII internalized less oAβ and AcLDL compared with SR-AI, whereas 341-exon11 internalized little oAβ or AcLDL (Additional file 3: Figure S2C,D and Figure 5G,H).

### The SRCR domain mediates the internalization of oAβ and AcLDL

**Figure 6:**
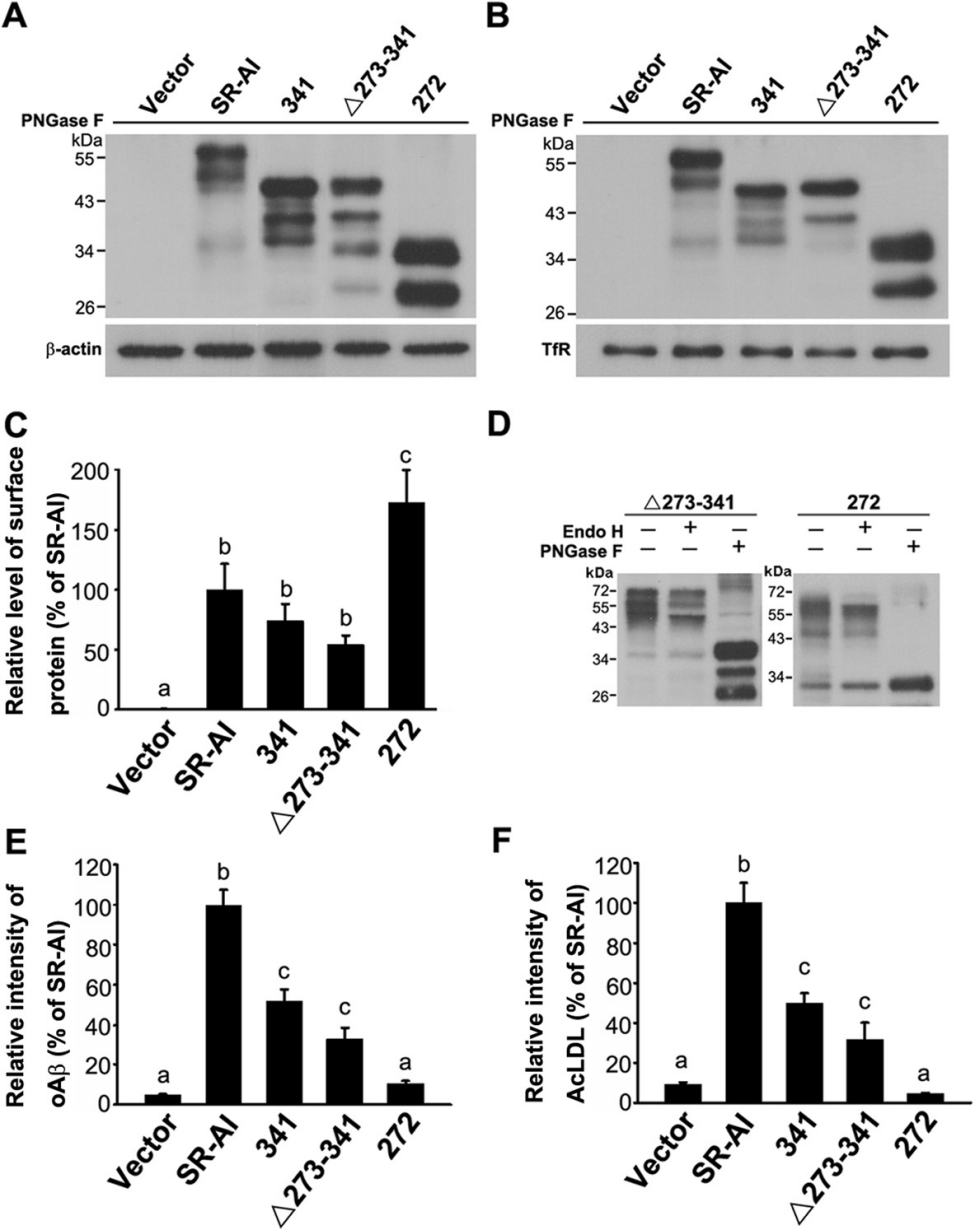
**Both SRCR and collagenous domains of SR-AI are oAβ- and AcLDL-binding domains. A** and **B**, Western blot analysis of total cell lysates and avidin pull-down of biotinylated lysates after PNGase F cleavage. **C**, Relative levels of surface-targeted SR-AI variants were quantified by densitometry. **D**, Western blot analysis of total lysates of transfected cells after PNGase F or Endo H cleavage. The experiments were repeated at least three times. **E** and **F**, Relative fluorescence intensities of internalized oAβ and AcLDL for more than 100 SR-AI variant-positive cells were quantified using MetaMorph software. Bars indicate mean ± SEM of three independent experiments. Experimental groups labeled with different letters were significantly different from each other (*p* < 0.05).

The collagenous domain has been identified as AcLDL binding domain [33]. Next, we examined whether the SRCR domain also mediates the ligand binding. Variants 341 and Δ273-341 lacked the SRCR and collagenous domain, respectively. Variant 272 lacked both the SRCR and collagenous domains. The protein level of 272 was higher than that of 341 and Δ273-341 in the total cell lysates (Figure 6A). The surface biotinylation assay and Western bolt analysis showed that all these deletion mutants were surface-targeted (Figure 6B). The densitometry analysis indicated similar surface protein levels of 341 and Δ273-341 (Figure 6C). Both Δ273-341 and 272 were predominately Endo H-resistant (Figure 6D). The surface-targeting of SR-AI, 341, and Δ273-341was further confirmed by the confocal images of surface-bound oAβ on the plasma membrane of SR-AI, 341, and Δ273-341-transfected cells (Additional file 4: Figure S3A). 341 and Δ273-341 internalized approximately 50% of the oAβ and AcLDL internalized by SR-AI (Additional file 4: Figure S3B,C and Figure 6E,F). These results indicated that the SRCR domain functioned as a binding domain for oAβ and AcLDL in the absence of the collagenous domain.

**Figure 7:**
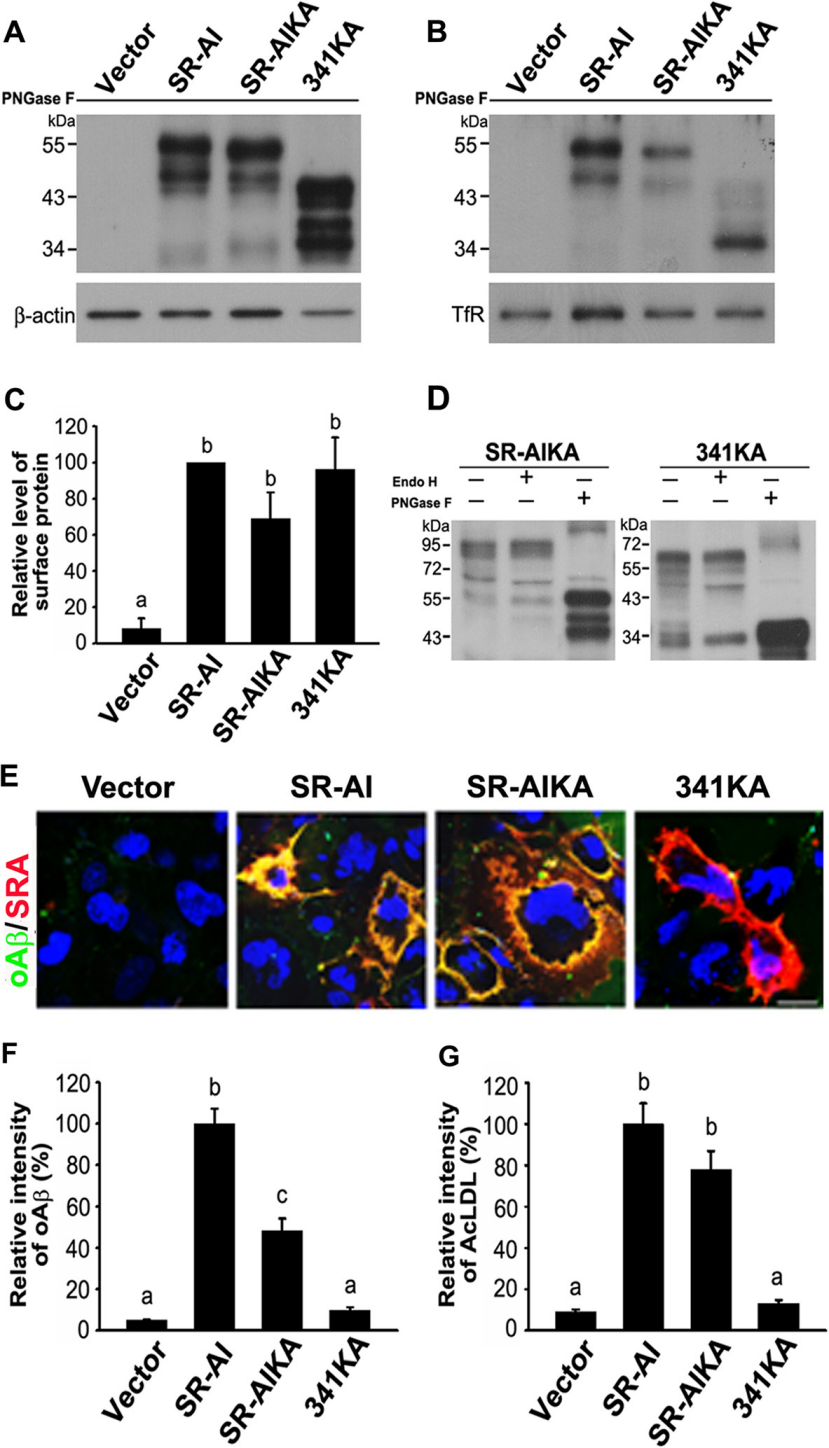
**The SRCR domain of SR-AIKA mediates the oAβ binding.** Lysine residues at 332, 335, and 338 in the collagenous domains of SR-AI and 341 were mutated to alanine. COS-7 cells were transfected with SR-AI, SR-AIKA, or 341KA. **A** and **B**, Western blot analysis of total cell lysates and avidin pull-down of biotinylated lysates after PNGase F cleavage. **C**, Relative levels of surface-biotinylated SR-AI variants were quantified. **D**, Western blot analysis of total cell lysates after PNGase F or Endo H cleavage. **E**, Representative merged confocal images of surface-bound oAβ on the plasma membrane of transfected cells. Scale bar, 20 μm. The experiments were repeated at least three times. **F** and **G**, Relative fluorescence intensities of internalized oAβ and AcLDL were quantified in more than 100 SR-A-positive cells using MetaMorph software. Bars indicate mean ± SEM of at least three independent experiments. Experimental groups labeled with different letters were significantly different from each other (*p* < 0.05).

Lysine residues at 332, 335, and 338 in the collagenous domain of SR-AI have been shown to be critical for AcLDL uptake [33]. We mutated three lysine residues at 332, 335, and 338 in SR-AI and 341. The internalization of oAβ and AcLDL by SR-AIKA and 341KA was examined. SR-AIKA and 341KA were surface-targeted and predominantly Endo H-resistant (Figure 7A-D). The live immunostaining showed that SR-AI, SR-AIKA, and 341KA were surface-targeted (red signal, Figure 7E). However, SR-AI and SR-AIKA, but not 341KA bound oAβ (yellow signal) at the plasma membrane. SR-AIKA internalized 50% oAβ and a similar amount of AcLDL compared to SR-AI (Figure 7F,G). However, 341KA internalized little oAβ and AcLDL. These data suggests that intact SRCR domain of SR-AIKA functions as a ligand binding domain while the ligand binding activity of the mutated collagenous domain was abolished.

## Discussion

In the present study, we use live immunostaining, surface biotinylation assay and ligand internalization to assess the functions of the SRCR domain of SR-AI. Our results provide the first evidence that the intact SRCR domain of SR-AI is critical for the protein folding and N-glycosylation. We show that the SRCR domain of SR-AI can serve as the binding domain of oAβ and AcLDL in the absence of the collagenous domain. The coimmunoprecipitation of BiP chaperon suggested that inefficiently folded intracellularly-retained SR-A variants were recognized by the endoplasmic reticulum-associated degradation pathway. These results thus indentify two novel functions of the intact SRCR domain which may be shared by the SRCR superfamily.

The functions of the SRCR domain of SR-AI were not identified by the previous studies due to several reasons. First of all, SR-AII lacking the SRCR domain is surface-targeted and binds AcLDL, suggesting that the SRCR domain may be not involved in AcLDL binding [34]. In addition, Doi et al. showed that a truncated variant of bovine SR-AI with a deletion of a long segment of the SRCR domain including exon 11 and part of exon 10 is surface-targeted and functional. However, mutants with partial or complete deletion of exon 11 were not available in their study [17]. Because they did not construct a plasmid containing an intact SRCR domain in the absence of the collagenous domain, the ligand-binding activity of the SRCR domain is not revealed in their study.

Cysteine residues in the SRCR domain, which form disulfide bonds, are involved in protein folding and resistance to biochemical and enzymatic stress [7,35]. Disulfide bond formation stabilizes and accelerates protein folding [36]. It is suggested that six cysteine residues in the SR-AI SRCR domain form three pairs of disulfide bonds. Variant 430 lacked two disulfide bonds, whereas variant 407 lacked all three disulfide bonds, suggesting that the truncated SRCR domain encoded by exon 11 may affect the folding of SR-AI protein. Electron microscopy revealed that a flexible hinge exists between the α-helix coiled-coil and the collagenous triple helix of SR-AI and SR-AII [37]. The angle of the two adjacent fibrous segments is zero degrees at physiological pH level, suggesting that SR-AI is compact, with the SRCR domain near the other domains. Therefore, the SRCR domain may be able to modulate protein folding of SR-AI because the hinge at zero degrees brings the SRCR domain close to the other domains.

It has been shown that misfolded proteins with exposed hydrophobic patches or unpaired cysteines elicit prolonged binding by the molecular chaperone BiP in the ER [32]. This prolonged BiP binding prevents the misfolded protein from being transported along the secretory pathway [38]. BiP also binds misfolded proteins bearing monoglycosylated N-glycans, which are beyond repair and released from the calnexin chaperone [32,39]. 341 was surface-targeted, however, the fusion of exon11 with 341 converted the compartment of this SR-AI variant into intracellular retention. The co-immunoprecipitation of BiP with suggests that 341-exon11 was not effectively folded. It is purposed that SR-AIII acts as a dominant negative regulator of SR-A activity to prevent excessive lipid uptake by macrophage and prevent form cell formation [40]. In the study, the ineffective folding of 341-exon11 which mimics SR-AIII provides a cellular mechanism for the intracellular retention of SR-AIII. The direct interaction of 341-exon11 and SR-AI was detected by co-immunoprecipitation (data not shown). Our findings suggest that SR-AI variants with truncated SRCR domain are detected by protein quality control mechanisms in the ER. Consistently, we found that the intracellular retention of SR-AI variants co-occurs with the arrest of N-glycosylation at high-mannose stages in cis-golgi.

## Conclusion

The SRCR domain is highly conserved within the SRCR superfamily with more than 30 proteins with diverse functions including pathogen recognition, and modulation of innate immunity. However, the roles of the SRCR domain of these proteins remain unclear. The novel functions of the SRCR domain of SR-AI revealed by this study may be conserved in the SRCR superfamily and provide opportunities to modulate the innate immunity and host defenses through regulating the level of protein surface targeting and ligand binding of SR-AI and members of the SRCR superfamily in microglia and macrophages.

## Abbreviations

SR-A: Scavenger receptor class A; MARCO: Macrophage receptor with collagenous structure; SRCR: Scavenger receptor cysteine-rich domain; oAβ: Oligomeric amyloid-β peptide; AcLDL: Acetylated low-density lipoprotein; TLR4: Toll-like receptor 4; AD: Alzheimer’s disease; PNGase F: Peptide N-glycosidase; Endo H: Endoglycosidase; ER: Endoplasmic reticulum.

## Competing interests

The authors declare that they have no competing interests.

## Authors’ contributions

FLH and YJS performed Western blot and the quantification analysis. CNY performed the functional analysis of microglia and macrophage. YJC constructed expression plasmids with deletion and point mutations. CHL performed tandem mass spectrometry analyses. SJH, FSS, and HJT prepared manuscripts. All authors read and approved the final manuscript.

## Supplementary Material

Additional file 1: Table S1 The primer sets and cloning strategy for expression constructs of SR-AI, SR-AII, and SR-AI variants. Click here for file

Additional file 2: Figure S1 Overexpressed human SR-AI in COS-7 is N-glycosylated and mediates the internalization of AcLDL and oAβ. **A**, Western blot analysis of total cell lysates of human macrophage and PMA-differentiated THP-1 cells and SR-AI-transfected COS-7 cells with and without PNGase F. **B**, Transfected COS-7 cells were incubated with Alexa 488-labeled AcLDL for 1 h at 37°C and immunostained with an anti-SR-A antibody. Representative confocal images showed that SR-AI-positive COS-7 cells internalized AcLDL (upper panel). The level of internalized AcLDL of SR-AI-positive or vector-positive cells was quantified by the flow cytometry. The mean fluorescence intensity of AcLDL uptake by SR-AI-positive COS-7 cells was significantly higher than the vector-positive COS-7 cells (lower panel). **C**, Transfected COS-7 cells were incubated with Alexa FAM-oAβ for 30 min at 37°C and immunostained with an anti-SR-A antibody. Representative confocal images showed that SR-AI-positive COS-7 cells internalized oAβ (upper panel). Relative fluorescence intensity of internalized oAβ of SR-AI-positive or vector-positive cells was quantified (lower panel). More than 100 SR-AI-positive cells in five random fields were analyzed. Bars indicate mean ± SEM of three different experiments (*p < 0.05). Nuclei were counterstained with Hoechst 33258 (blue). Scale bar, 20 μm. Click here for file

Additional file 3: Figure S2 Internalization of oAβ and AcLDL by COS-7 cells transfected with SR-AI and variants. Cells were incubated with FAM-oAβ for 30 min or Alexa 594-labeled AcLDL for 1 h at 37°C. Cells were immunostained with an anti-SR-A antibody. **A** and **B**, SR-AI and variants 371 and 341 internalized oAβ and AcLDL. Variants 430 and 407 failed to internalize oAβ and AcLDL. **C** and **D**, SR-AI- and SR-AII- but not 341-exon11-positive cells internalized oAβ and AcLDL. Nuclei were counterstained with Hoechst 33258 (blue). Scale bar, 20 μm. Nuclei were counterstained with Hoechst 33258 (blue). Scale bar, 20 μm. Click here for file

Additional file 4: Figure S3 Both SRCR and collagenous domains bind and internalize oAβ and AcLDL. COS-7 cells were transfected with SR-AI, 341, Δ273-341, and 272. **A**, Representative confocal images of surface-bound oAβ on the plasma membrane of transfected cells. Yellow signal of merged images represented the surface-targeted SR-AI, 341, and Δ273-341 bind oAβ (green) at the plasma membrane as shown by SR-A live immunostaining (red). Scale bar, 20 μm. **B** and **C**, SR-AI, 341, and Δ273-341-positive cells internalized oAβ and AcLDL. Nuclei were counterstained with Hoechst 33258 (blue). Scale bar, 20 μm. Click here for file
