# Supplementary figures and images for: Cysteine-rich domain of scavenger receptor AI modulates the efficacy of surface targeting and mediates oligomeric Aβ internalization
Source: J Biomed Sci. 2013 Aug 2;20(1):54. doi: 10.1186/1423-0127-20-54 (PMC3750411; doi:10.1186/1423-0127-20-54)

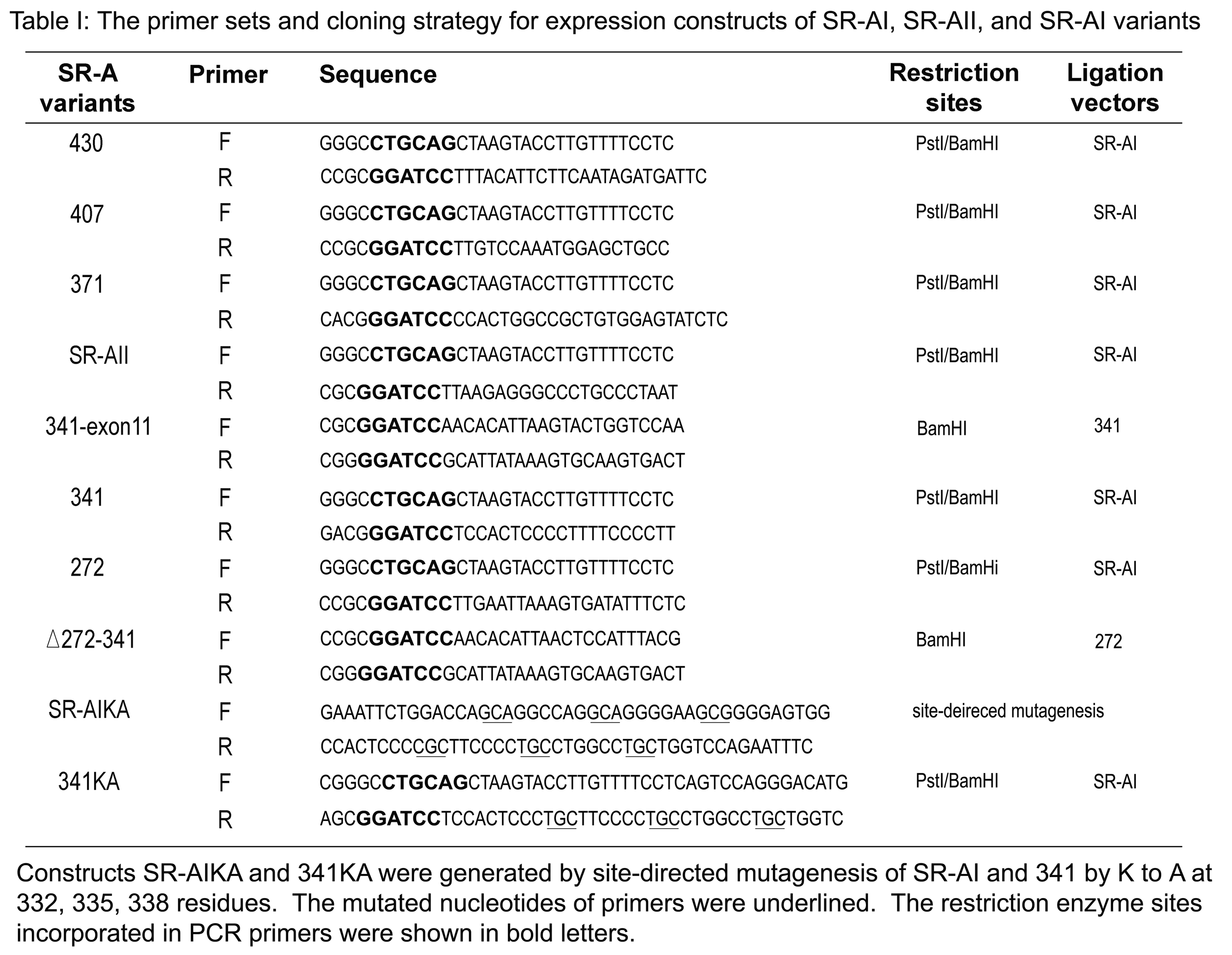

Supplement: Additional file 1: Table S1 — The primer sets and cloning strategy for expression constructs of SR-AI, SR-AII, and SR-AI variants. [file 1423-0127-20-54-S1.tiff]

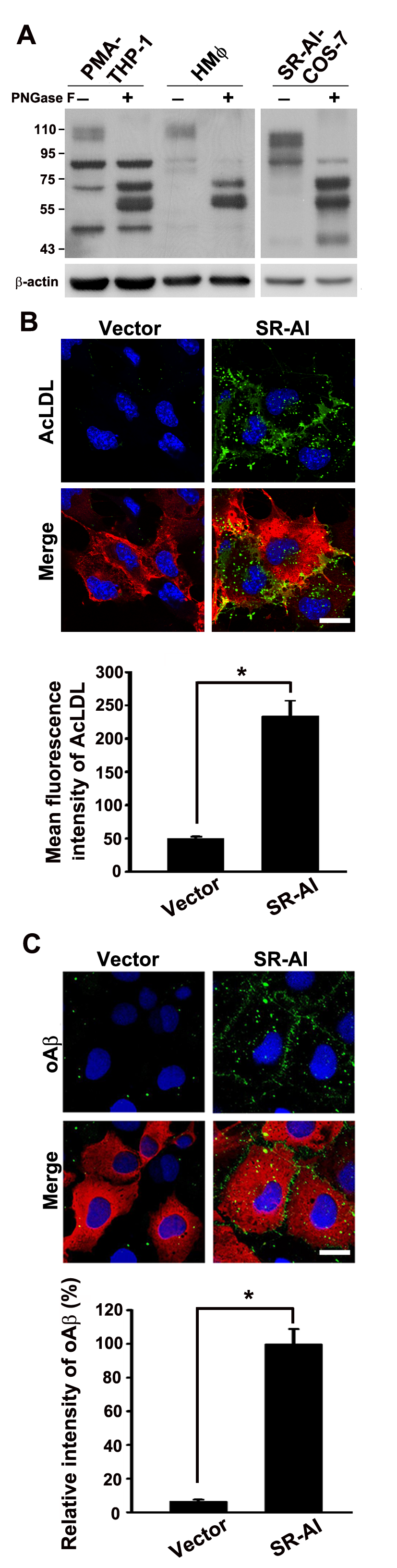

Supplement: Additional file 2: Figure S1 — Overexpressed human SR-AI in COS-7 is N-glycosylated and mediates the internalization of AcLDL and oAβ. A, Western blot analysis of total cell lysates of human macrophage and PMA-differentiated THP-1 cells and SR-AI-transfected COS-7 cells with and without PNGase F. B, Transfected COS-7 cells were incubated with Alexa 488-labeled AcLDL for 1 h at 37°C and immunostained with an anti-SR-A antibody. Representative confocal images showed that SR-AI-positive COS-7 cells internalized AcLDL (upper panel). The level of internalized AcLDL of SR-AI-positive or vector-positive cells was quantified by the flow cytometry. The mean fluorescence intensity of AcLDL uptake by SR-AI-positive COS-7 cells was significantly higher than the vector-positive COS-7 cells (lower panel). C, Transfected COS-7 cells were incubated with Alexa FAM-oAβ for 30 min at 37°C and immunostained with an anti-SR-A antibody. Representative confocal images showed that SR-AI-positive COS-7 cells internalized oAβ (upper panel). Relative fluorescence intensity of internalized oAβ of SR-AI-positive or vector-positive cells was quantified (lower panel). More than 100 SR-AI-positive cells in five random fields were analyzed. Bars indicate mean ± SEM of three different experiments (*p < 0.05). Nuclei were counterstained with Hoechst 33258 (blue). Scale bar, 20 μm. [file 1423-0127-20-54-S2.tiff]

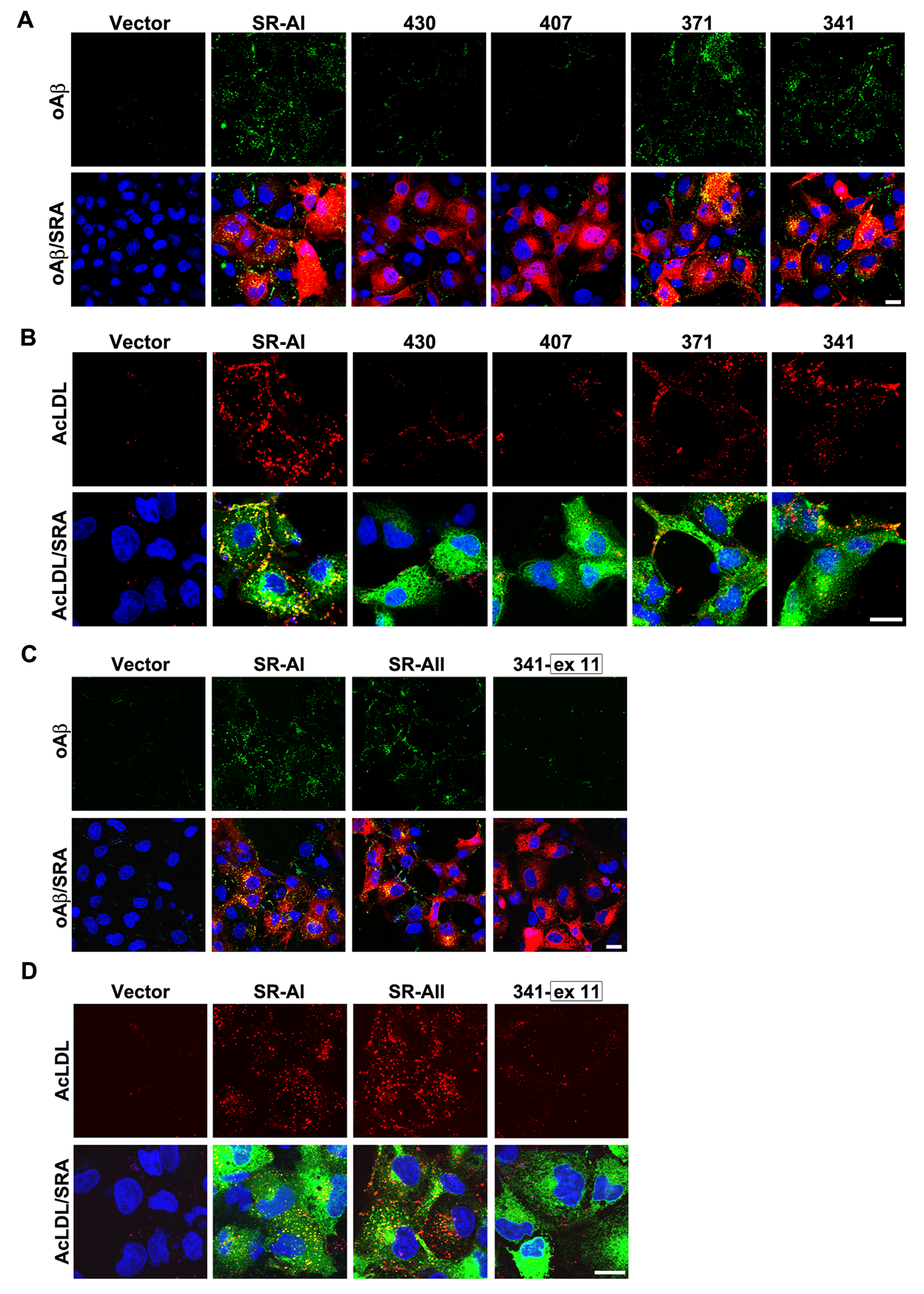

Supplement: Additional file 3: Figure S2 — Internalization of oAβ and AcLDL by COS-7 cells transfected with SR-AI and variants. Cells were incubated with FAM-oAβ for 30 min or Alexa 594-labeled AcLDL for 1 h at 37°C. Cells were immunostained with an anti-SR-A antibody. A and B, SR-AI and variants 371 and 341 internalized oAβ and AcLDL. Variants 430 and 407 failed to internalize oAβ and AcLDL. C and D, SR-AI- and SR-AII- but not 341-exon11-positive cells internalized oAβ and AcLDL. Nuclei were counterstained with Hoechst 33258 (blue). Scale bar, 20 μm. Nuclei were counterstained with Hoechst 33258 (blue). Scale bar, 20 μm. [file 1423-0127-20-54-S3.tiff]

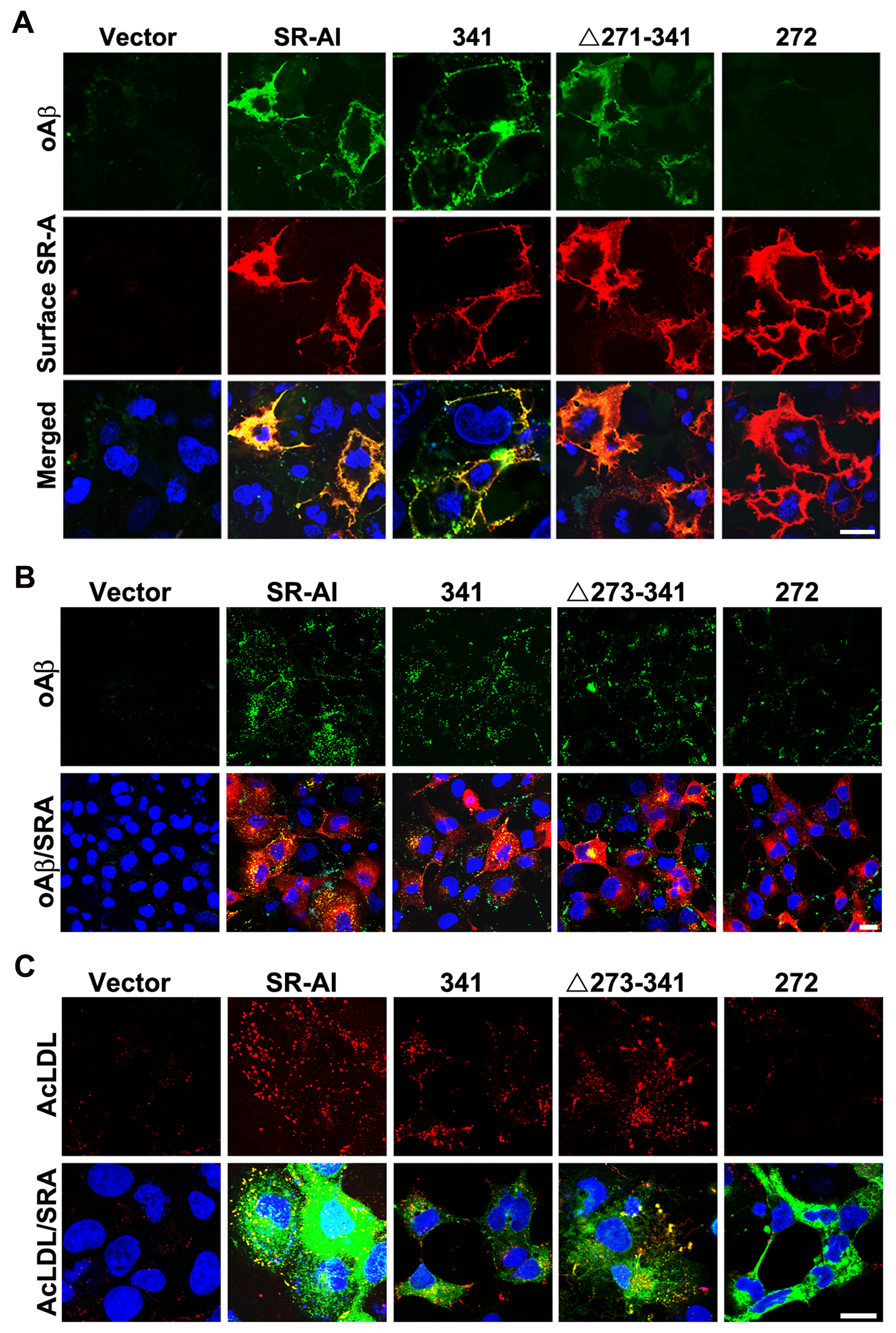

Supplement: Additional file 4: Figure S3 — Both SRCR and collagenous domains bind and internalize oAβ and AcLDL. COS-7 cells were transfected with SR-AI, 341, Δ273-341, and 272. A, Representative confocal images of surface-bound oAβ on the plasma membrane of transfected cells. Yellow signal of merged images represented the surface-targeted SR-AI, 341, and Δ273-341 bind oAβ (green) at the plasma membrane as shown by SR-A live immunostaining (red). Scale bar, 20 μm. B and C, SR-AI, 341, and Δ273-341-positive cells internalized oAβ and AcLDL. Nuclei were counterstained with Hoechst 33258 (blue). Scale bar, 20 μm. [file 1423-0127-20-54-S4.tiff]
